# Mfsd2a attenuated hypoxic*-*ischemic brain damage via protection of the blood–brain barrier in* mfat-1* transgenic mice

**DOI:** 10.1007/s00018-023-04716-9

**Published:** 2023-02-23

**Authors:** Xiaoxue Li, Yumeng Zhang, Jianghao Chang, Chenglin Zhang, Lin Li, Yifan Dai, Haiyuan Yang, Ying Wang

**Affiliations:** 1 Department of Medical Genetics, School of Basic Medical Science, Nanjing Medical University, Nanjing, 211166 China; 2 Jiangsu Key Laboratory of Xenotransplantation, Nanjing Medical University, Nanjing, 211166 China; 3 Key Laboratory of Targeted Intervention of Cardiovascular Disease, Collaborative Innovation Center for Cardiovascular Disease Translational Medicine, Nanjing Medical University, Nanjing, 211166 China; 4 State Key Laboratory of Reproductive Medicine, Nanjing Medical University, Nanjing, 211166 China

**Keywords:** NormFinder, LC–MS/MS, Hepatocytes, PLA1, TEM, Apoptosis

## Abstract

Previous studies have shown that *mfat-1* transgenic mice have protective effects against some central nervous system (CNS) disorders, owing to the high docosahexaenoic acid (DHA) content enriched in their brains. However, whether this protective effect is connected to the blood*–*brain barrier (BBB) remains unclear. This study aims to investigate the mechanisms of the protective effect against hypoxic-ischemic brain damage (HIBD) of *mfat-1* transgenic mice. *mfat-1* mice not only demonstrated a significant amelioration of neurological dysfunction and neuronal damage but also partly maintained the physiological permeability of the BBB after HIBD. We initially showed this was associated with elevated major facilitator superfamily domain-containing 2a (Mfsd2a) expression on the BBB, resulting from more lysophosphatidylcholine (LPC)-DHA entering the brain. Wild-type (WT) mice showed a similar Mfsd2a expression trend after long-term feeding with an LPC-DHA-rich diet. Knockdown of Mfsd2a by siRNA intra-cerebroventricular (ICV) injection neutralized the protective effect against HIBD-induced BBB disruption in *mfat-1* mice, further validating the protective function of Mfsd2a on BBB. HIBD-induced BBB high permeability was attenuated by Mfsd2a, primarily through a transcellular pathway to decrease caveolae-like vesicle-mediated transcytosis. Taken together, these findings not only reveal that *mfat-1* transgenic mice have higher expression of Mfsd2a on the BBB, which partly sustains BBB permeability via vesicular transcytosis to alleviate the severity of HIBD, but also suggest that dietary intake of LPC-DHA may upregulate Mfsd2a expression as a novel therapeutic strategy for BBB dysfunction and survival in HIBD patients.

**Supplementary Information:**

The online version contains supplementary material available at 10.1007/s00018-023-04716-9.

## Introduction

Hypoxic-ischemic (HI) brain damage (HIBD) occurs when cerebral blood flow is decreased or suspended, resulting in partial or complete hypoxia of brain tissues [1]. In neonates and adults, brain injury produced by HI is recognized as a devastating incident that frequently leads to death or profound long-term neurological morbidity [2]. Perinatal asphyxia is the leading cause of HIBD in neonates, affecting three out of every 1000 term newborns [3]. Adults have seen an increase in the prevalence of brain ischemia in recent years due to cardiac arrest or cerebrovascular illness, with subsequent hypoxia caused by decreased blood flow [4]. As a result of the preceding, HIBD has emerged as one of the most severe public health issues. While studies on HIBD are becoming increasingly in-depth, the underlying mechanisms are challenging to decipher and remain largely elusive. Meanwhile, there are also no FDA-approved pharmacotherapies for the treatment of HIBD [5]. Therefore, it is urgent to explore an effective way to prevent or alleviate HIBD.

The blood–brain barrier (BBB) is a selectively semipermeable boundary formed by central nervous system (CNS) vascular endothelial cells (ECs) [6]. Two primary mechanisms of ECs maintain the low permeability of the BBB: (i) by suppressing vesicular trafficking or transcytosis (transcellular pathway) [7]; (ii) by limiting intercellular transit via specialized tight junctions (TJ) complexes (paracellular pathway). BBB breakdown has been linked to various acute and chronic CNS diseases, highlighting the possible deleterious implications of BBB disruption on brain function [8]. Major facilitator superfamily domain-containing 2a (Mfsd2a) expresses predominantly in the cerebral microvessel endothelium and serves as the primary membrane transporter for lysophosphatidylcholine (LPC)-DHA uptake [9]. Additionally, Mfsd2a is a crucial regulator of BBB function [10], and it is unknown whether Mfsd2a contributes to BBB disruption following HI.

Previous studies have established that *fat-1* transgenic mice can protect against various CNS disorders [11]. The *mfat-1* (mammalian *fat*-*1,* thereafter called *mfat*-*1*) transgenic mice, overexpressing a codon-optimized ω-3 desaturase derived from *C*. *elegans*, can synthesize ω-3 polyunsaturated fatty acids (PUFAs) from ω-6 PUFAs, thereby maintaining a low and well-balanced ratio of ω-6/ω-3 PUFAs in their tissues [12]. It is worth mentioning that the brain of *mfat*-*1* mice has a significantly higher level of total DHA, the main component of ω-3 PUFAs [13, 14]. DHA cannot be de novo synthesized in the brain and must be imported across the BBB from plasma [15, 16]. In the liver, some enzymes, especially hepatic lipase, can act preferentially on phospholipids containing DHA at the sn-2 position, resulting in LPC-DHA [17]. Mfsd2a*-*deficient mice show a noticeable drop in cerebral LPC-DHA levels [18], indicating that Mfsd2a is the primary transporter of LPC-DHA into the brain [9]. Furthermore, increasing the expression of genes encoding fatty acid transporters increases PUFA uptake [19–21]. Hence, we speculated that when more LPC-DHA derived from plasma was required for transport into the brain of *mfat-1* mice, the expression of Mfsd2a protein on the BBB would increase accordingly.

Currently, few studies have described the molecular mechanism underlying *mfat*-*1* transgenic mice's resistance to HIBD. In our study, we found that *mfat-1* mice partially maintained BBB physiological function following HIBD. We showed that the Mfsd2a expression on the BBB was upregulated in both WT mice fed with LPC-DHA-rich diet and *mfat-1* mice, mainly associated with more LPC-DHA entering the brain. In addition, we proved that the elevated Mfsd2a in *mfat-1* mice could attenuate HIBD-induced BBB breakdown primarily through the transcellular pathway to reduce caveolae-like vesicles-mediated transcytosis. These findings revealed the protective effect and underlying molecular mechanisms of *mfat-1* transgenic mice against HIBD, which will provide insight into future research and development of safe and effective HIBD prevention and treatment programs.

## Results

### *mfat*-*1* mice partially reversed HIBD-induced neurological dysfunction

The triphenyl-2,3,5-tetrazolium-chloride (TTC) staining and T2WI of magnetic resonance imaging (MRI) were used to determine the effectiveness of the mouse HIBD model, both of which showed massive cerebral infarction (the former, stained white; the latter, hyperintense) in the left cortical region of the wild-type (WT) mouse after HIBD (Fig. S1a, b). There was distinct cerebral edema in the left hemisphere, resulting in asymmetry of both cerebral hemispheres and the brain midline shifting (Fig. S1b). In addition, we found the body of the mice was apparently tilting to the left (affected side) (Fig. S1c), the ipsilateral eye remained almost closed (Fig. S1d), and cerebral ischemic infarction accompanied by edema (Fig. S1e) after the operation. Severe symptomatic mice died within a short time in our study.

**Fig. 1:**
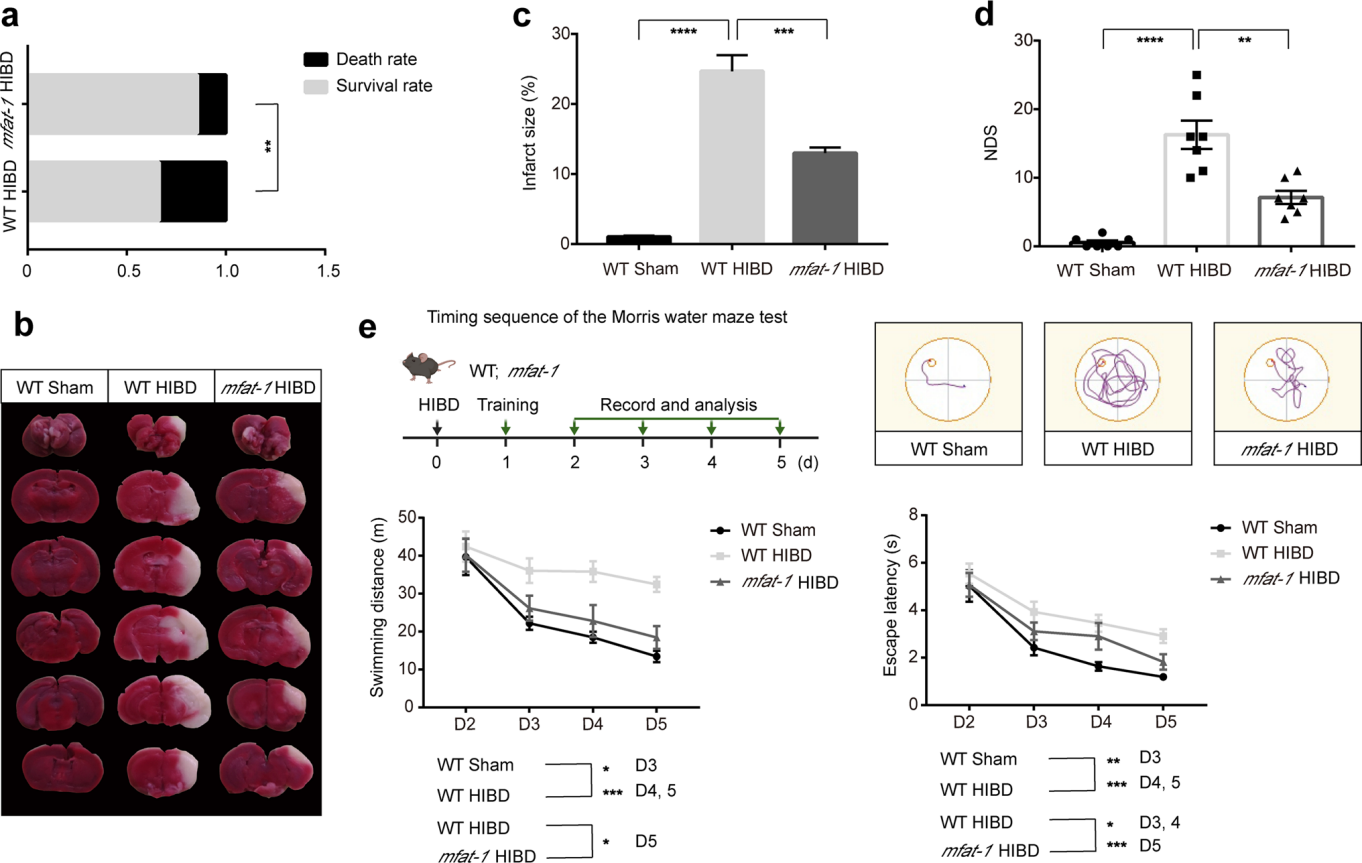
*mfat*-*1* mice alleviated the HIBD-induced neurological dysfunction. **a** Respective rate of death and survival in the WT (*n* = 83) and *mfat*-*1* (*n* = 90) groups within one day post-HIBD surgery. **b**, **c** Representative images of TTC-stained cerebral coronal sections of mice after HIBD in the WT, *mfat*-*1* group, Sham-operation group (**b**), and quantitative assessment of cerebral infarction volume in each group (**c**) (*n* = 7). **d** The NDS determination in three groups (*n* = 7). **e** Up: timing sequence of the Morris water maze test and representative tracing images in each group. Down: swimming distance and escape latency were shortened in *mfat*-*1* mice post-HIBD (*n* = 12). *p < 0.05, **p < 0.01, ***p < 0.001, ****p < 0.0001

To determine whether the *mfat*-*1* mice could reduce mortality following HIBD, we calculated the death and survival rates within one day post-operation. Compared with the WT HIBD group, a significantly reduced death rate and a corresponding increase in the survival rate were observed in the *mfat-1* HIBD group (Fig. 1a). As the cerebral infarction volume in mice was a critical indicator for the severity of HIBD, we performed TTC staining to show ischemic infarction, which was considerably diminished in the *mfat*-*1* HIBD mice than in the WT HIBD mice (Fig. 1b, c). The neurological deficit scores (NDS) determination is used to assess the degree of neurological deficits proportional to the severity [22]. The results showed NDS in the *mfat*-*1* HIBD group was lower than in the WT HIBD group, indicating less severe neurological impairments (Fig. 1d). It is well established that HIBD impairs spatial learning and memory [23]. The Morris water maze was adopted to assess the spatial learning performance, which revealed that *mfat-1* HIBD mice spent less time finding the hidden platform underneath the water than their WT counterparts during the acquisition and training phase, as exhibited by swimming distance and escape latency values in the experiments (Fig. 1e). The *mfat-1* mice appeared to have a protective effect against HIBD*-*induced neurological dysfunction.

### *mfat*-*1* mice were protective against neuronal damage post-HIBD

**Fig. 2:**
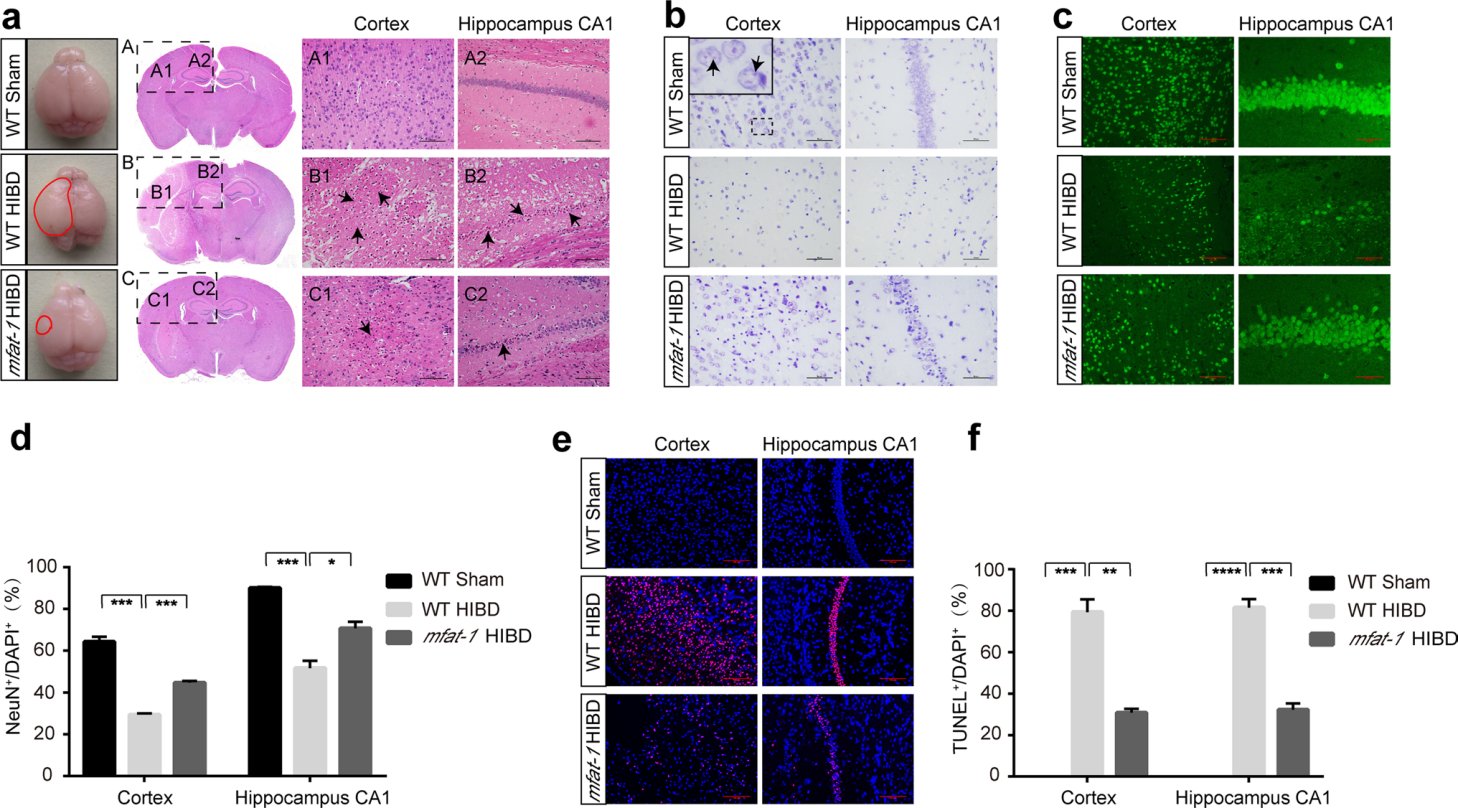
HIBD-induced neuronal damage was attenuated in the cerebral cortex and hippocampus CA1 of *mfat-1* mice. **a** Left: overall view of the whole brain of mice in the WT Sham, WT HIBD, and *mfat*-*1* HIBD groups. The solid red lines represent the cortical ischemic infarct area observed by the naked eye. Right and **b** H&E and Nissl's staining showed HIBD-induced neuronal damage in the left cortex and hippocampus CA1 area in each group. The black arrows indicate injured neurons (**a**). The solid black box is an enlargement of the black dashed box. The black arrows represent the Nissl bodies within the neuron, which are the main sites of protein synthesis in the neurons (**b**). Scale bar, 50 μm. **c and d** Anti-NeuN immunofluorescence staining in the left cortex and hippocampus CA1 of coronal brain sections indicated decreased mature neurons post-HIBD (**c**). Scale bars, 100 μm (left); 50 μm (right). Quantitative analysis of the ratio of NeuN^+^/DAPI^+^ cells showed that *mfat*-*1* HIBD mice had lower neuronal loss than WT HIBD mice (**d**) (*n* = 7). **e and f** Representative images of TUNEL staining in the left cerebral cortex and hippocampus CA1 of the brain after HIBD in each group (**e**), and the quantification results of the percentage of TUNEL^+^/DAPI.^+^ cells (**f**) (*n* = 7). Scale bar, 100 μm. H&E, hematoxylin and eosin; *p < 0.05, **p < 0.01, ***p < 0.001, ****p < 0.0001

Cerebral HI can trigger acute neuron death, morphological change, and neuroinflammation [24, 25]. Among them, neuronal cell death is a crucial contributor to neurologic deficits after HIBD [26]. We selected the cortical infarcted area and hippocampal CA1 for analysis, for the pyramidal neurons of CA1 are the most affected cells after ischemic brain damage [27]. Histopathological analysis showed no marked morphological change in neurons in the WT Sham group (Fig. 2a). In contrast, significant morphological abnormalities, including neuronal cell loss, nuclei shrinkage, and empty cell formation, were observed in the infarct area and hippocampal CA1 of HIBD mice. Noticeably, the above phenomenon was significantly ameliorated in the *mfat-1* HIBD group (Fig. 2a, c, d). As revealed by Nissl's staining, there is a marked reduction in the numbers of neurons and Nissl bodies in WT mice but not in *mfat*-*1* animals, suggesting a protective effect of *mfat*-*1* on neurons against HIBD (Fig. 2b). The immunofluorescence results of neuronal nuclei (NeuN, a neuronal marker) showed a similar pattern. Compared to Sham animals, the ratio of NeuN-positive to DAPI-positive cells was diminished in both WT HIBD and *mfat*-*1* HIBD mice, while the latter displayed a much lesser extent (Fig. 2c, d). As the ability of learning and memory is associated with the number of neuronal cells [28, 29], we reasoned that the HIBD-induced neurological impairment shown in Fig. 1e was caused by a loss in neurons.

We continued to evaluate neuronal apoptosis to further examine the positive impact of *mfat*-*1* mice after HIBD. Limited numbers of TUNEL-positive neurons were detected in Sham mice, while a markedly higher ratio of TUNEL-positive to DAPI-positive cells was found in WT HIBD mice than in *mfat*-*1* HIBD animals (Fig. 2e, f). The immunofluorescence results of cleaved caspase 3 (an apoptosis marker) showed the same trend as above (Fig. S2a, b). These findings indicated that *mfat*-*1* mice exerted a protective effect against HIBD-induced neurological dysfunction, neuronal damage, and poor functional outcome, which may be an inspiration for alleviating HIBD and promoting neuron survival to improve HIBD sequelae [30].

### The physiological permeability of BBB was maintained in part in *mfat*-*1* mice after HIBD

**Fig. 3:**
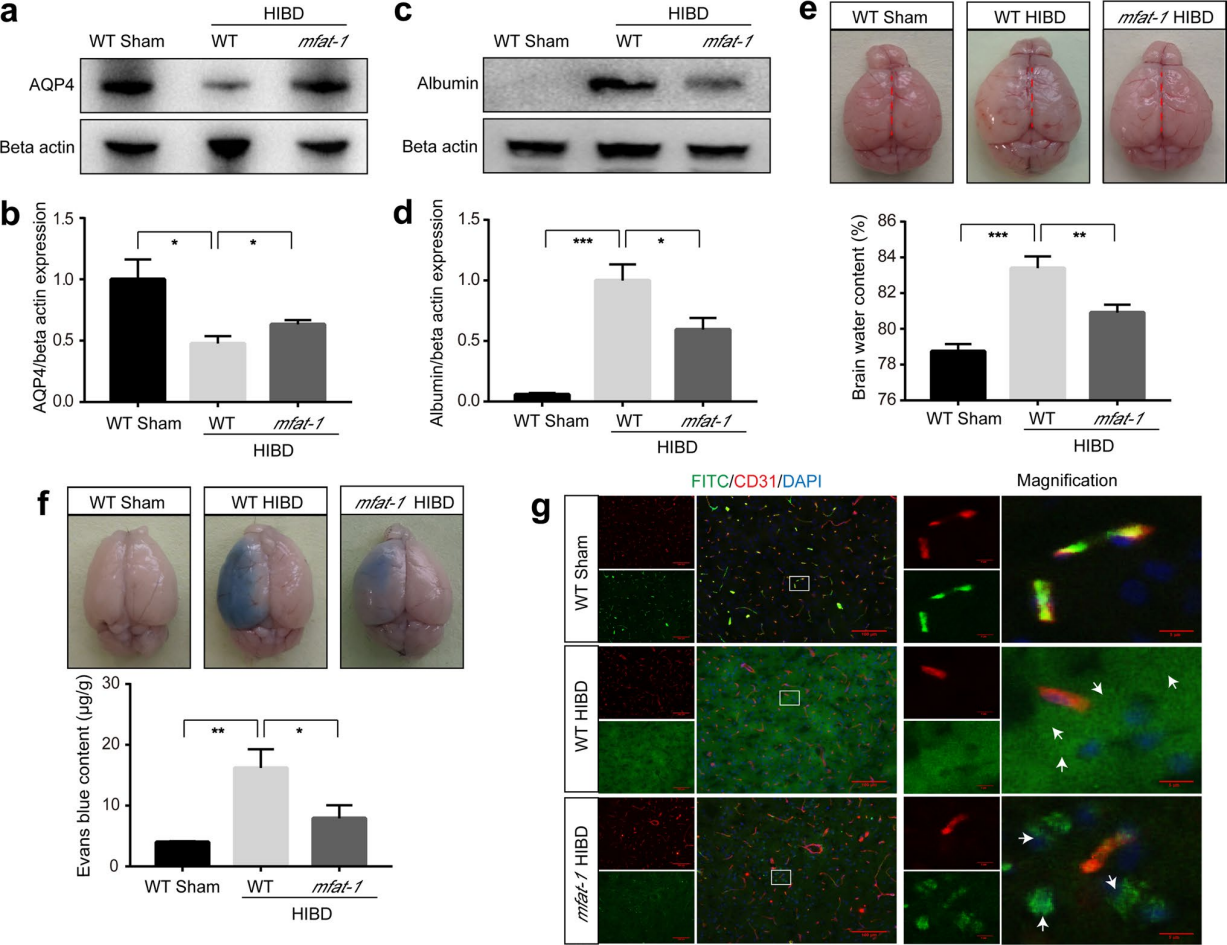
Reduced impairment to the permeability of BBB in *mfat*-*1* mice after HIBD. **a-d** Representative western blots showed the expression of AQP4 and albumin proteins in the WT Sham, WT HIBD, and *mfat*-*1* HIBD groups (**a**, **c**) and quantification analysis (**b**, **d**) (*n* = 7). **e** Percentage of the brain water content was evaluated at one day post-HIBD in the three groups. The animals in the WT HIBD exhibited severe cerebral edema owing to disrupted BBB. The red dotted lines represent the midline of the brain. Compared with their right cerebral hemisphere, the affected hemisphere of HIBD mice showed edema (*n* = 7). **f** EB accumulation in the affected hemisphere was visible macroscopically in the WT HIBD and *mfat*-*1* HIBD mice. Quantification of EB content revealed a significant decrease in EB leakage in the *mfat*-*1* HIBD mice compared to that in the WT HIBD mice (*n* = 7). **g** The dextran (green) was confined to the capillaries (marked by CD31, red) in WT Sham mice, and the *mfat*-*1* HIBD mice leaked less dextran (white arrows) from blood vessels, suggesting lower BBB permeability than WT HIBD mice. The enlarged part on the right was the position of the solid white line on the left. Scale bar, 100 μm. *p < 0.05, **p < 0.01, ***p < 0.001

The ensuing vasogenic cerebral edema is one of the major complications after the occurrence of HIBD due to disruption of the BBB [31, 32], which is a life-threatening event. Aquaporin 4 (AQP4), the major AQP in the brain, mediates free transmembrane transport of water molecules, as evidenced by decreased expression in vasogenic cerebral edema (BBB incomplete) [33]. As expected, AQP4 expression in the brain of WT HIBD mice was significantly reduced compared to the *mfat*-*1* HIBD group (Fig. 3a, b). Similarly, the brain water content was significantly decreased in *mfat*-*1* mice following HIBD compared with WT, with the lowest in the Sham group (Fig. 3e). BBB disruption enhanced injury progression during and after ischemic stroke [34]. Analysis of circulating albumin protein (65 kDa) also revealed a disrupted BBB because albumin was barely detectable in the healthy brain. *mfat*-*1* mice showed decreased albumin entry into the brain parenchyma after HIBD, suggesting an improvement in the extent of BBB disruption (Fig. 3c, d).

To further determine the functional integrity of the BBB in vivo, we monitored vascular permeability after injecting intravenously either Evans Blue (EB) dye or FITC-conjugated tracer (dextran, 40 kDa), which usually cannot cross the intact BBB (Fig. 3f, g) [35, 36]. EB can not only bind to plasma albumin to turn into a high molecular weight permeability marker (69 kDa) once it enters the bloodstream [37], but it can also accurately demonstrate the zone of altered permeability in specific brain regions with BBB breakdown (Fig. 3f). Our results indicated a disrupted BBB after HIBD, as revealed by EB (Fig. 3f) and dextran (Fig. 3g) extravasation from blood vessels into the brain parenchyma. Compared to the WT HIBD mice, *mfat-1* HIBD mice showed a lower extent of EB or dextran leakage, implying *mfat-1* mice exhibited a decreased permeability of the BBB following HIBD (Fig. 3f, g).

### Elevated Mfsd2a expression in *mfat-1* transgenic mice

**Fig. 4:**
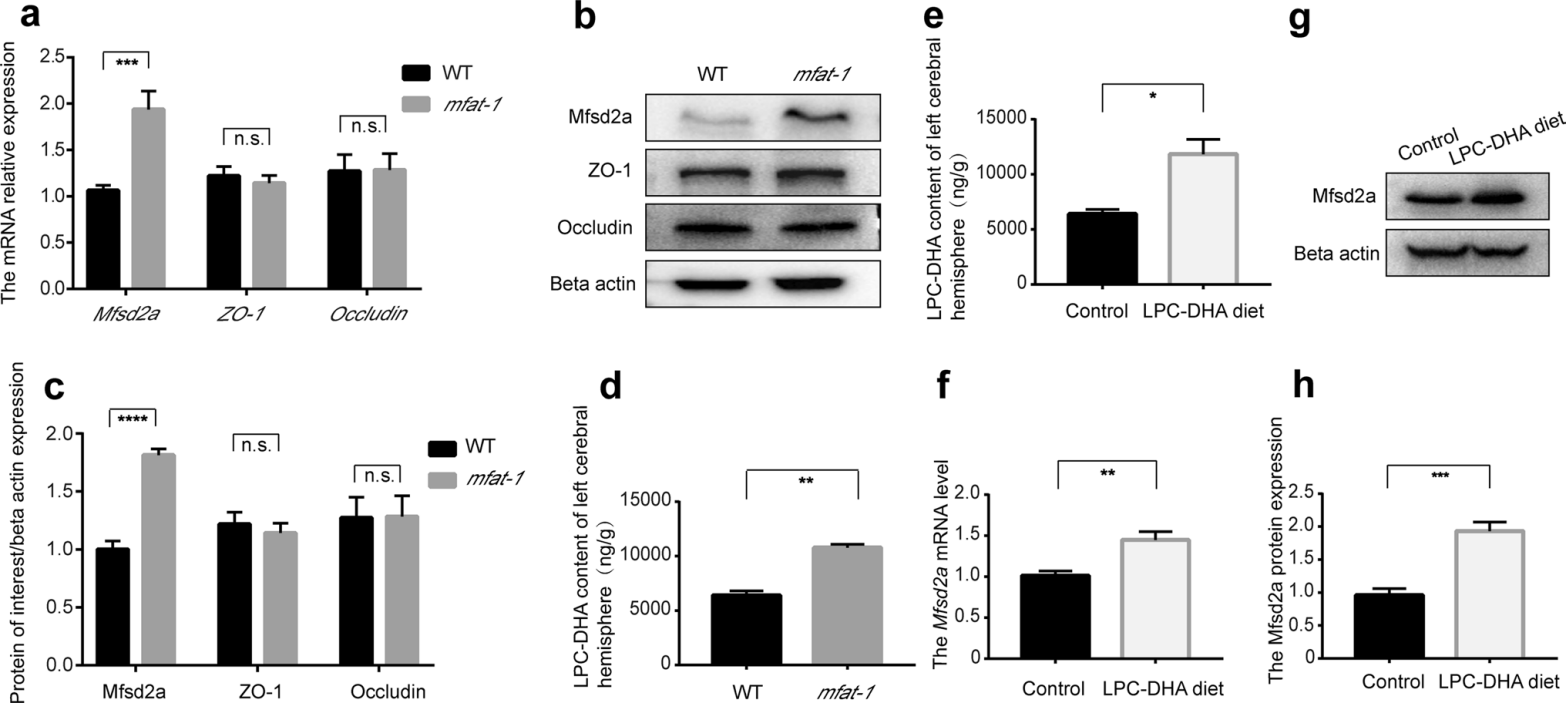
Mfsd2a expression was upregulated in the brain of WT mice fed with LPC-DHA-rich diet or *mfat-1* mice. **a** The mRNA abundance of *Mfsd2a*, *ZO-1,* and *occludin* normalized to the *beta actin* gene in WT and *mfat-1* mice. The *Mfsd2a* mRNA expression in the left brain of *mfat-1* mice was ~ two fold higher than that of WT mice. But there was no statistical difference in the expression of *ZO*-*1* and *occludin* (TJ-related genes) (*n* = 7). **b** Representative images about the expression of Mfsd2a, ZO-1, and occludin in the left brain of WT and *mfat*-*1* mice by western blot. **c** The band intensity of Mfsd2a, ZO-1, and occludin relative to beta actin was detected, respectively. No significant change was observed in the ZO-1 and occludin protein levels in WT and *mfat-1* mice (*n* = 7). **d** The LPC-DHA content of the left cerebral hemisphere in WT and *mfat-1* mice was determined by LC–MS/MS (*n* = 3). **e** LPC-DHA content of the left cerebral hemisphere in the control (regular diet) group and LPC-DHA- rich diet group through LC–MS/MS (*n* = 3). **f**–**h** The Mfsd2a level was presented by qRT-PCR (**f**) and western blot (**g**, **h**) in the control group and LPC-DHA-rich diet group (*n* = 7). *n.s.* no significance; *p < 0.05, **p < 0.01, ***p < 0.001, ****p < 0.001

BBB dysfunction is a prominent pathological feature of CNS disorders and is typically associated with poor outcomes [38–40]. The paracellular and transcellular pathways act cooperatively to sustain the normal physiological permeability of the BBB. The former includes occludin, zonula occluden-1 (ZO-1), claudins, and so on [41, 42], and Mfsd2a belongs to the latter [7]. We initially investigated the expression of Mfsd2a, ZO-1, and occludin in the WT and *mfat*-*1* mice by RT-qPCR and western blot, finding that the Mfsd2a expression was substantially higher in the brain of *mfat-1* mice than in WT mice (Fig. 4a–c). However, there was no significant difference in ZO-1 and occludin mRNA and protein levels between the WT and the *mfat*-*1* mice (Fig. 4a–c).

Previous results showed that the DHA content was significantly higher in the liver and brain of *mfat*-*1* transgenic mice [43]. DHA in the brain is supplied from the liver through blood circulation, containing unesterified DHA and LPC-DHA. The optimal form of DHA for brain uptake is LPC via the specific transporter, Mfsd2a [9]. Liquid chromatography-tandem mass spectrometry (LC–MS/MS), a common method for absolutely quantifying fatty acids [44], showed a significant increase in the brain's LPC-DHA content in *mfat-1* mice (Fig. 4d). To further verify whether the peripheral increased LPC-DHA entering the brain resulted in elevated Mfsd2a expression on the BBB of *mfat-1* mice, we fed WT mice with LPC-DHA-rich diet for 3 months. Notably, compared with the regular diet group, the levels of LPC-DHA and Mfsd2a in the brain in the LPC-DHA diet group presented the same trend, an upward trend (Fig. 4e-h). The results mentioned above raised the hypothesis that more LPC-DHA entering the brain led to an enhanced expression of transporter—Mfsd2a on the BBB. We reasoned that the higher expression level of Mfsd2a in the brain of *mfat*-*1* mice is related to the increased endogenous LPC-DHA content in *mfat*-*1* mice.

### The expression of Mfsd2a was altered by HIBD

**Fig. 5:**
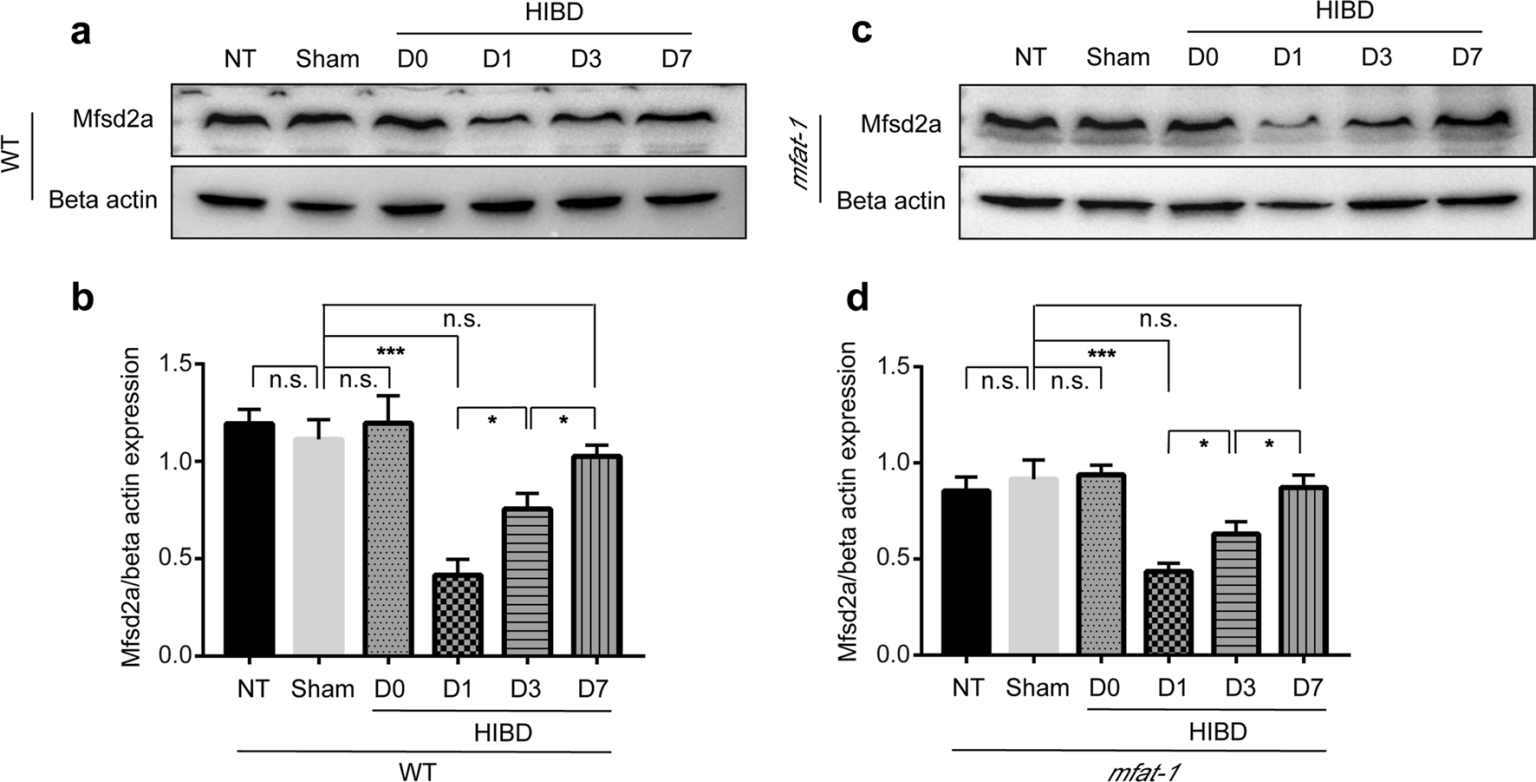
Mfsd2a expression was at the lowest level on the day post-HIBD. **a**–**d** Mfsd2a protein expression changed under different experimental conditions, and the band intensity of Mfsd2a was normalized to beta actin in WT (**a**, **b**) and *mfat-1* (**c**, **d**) mice (*n* = 7). *NT* no treated; *n.s.* no significance; *p < 0.05, ***p < 0.001

Certain CNS disorders, such as subarachnoid hemorrhage, have been linked to BBB disruption due to Mfsd2a deficiency [35]. Compared to the Sham group, Mfsd2a expression in WT HIBD mice decreased considerably in the damaged cerebral hemisphere, peaked at one day post-HIBD, and then gradually regained (Fig. 5a, b). The same characteristic was also shown in *mfat-1* mice (Fig. 5c, d). These results suggested that Mfsd2a might be involved in or trigger the disruption of the BBB following HIBD. The expression pattern of Mfsd2a after HIBD varied throughout time, and day one was an optimal time point to assess BBB-related indicators. Based on this finding, the follow-up experiments were carried out in mice one day following surgery as the appropriate time point for detection and analysis. Mfsd2a may be associated with the protection against HIBD-induced BBB dysfunction from the results given in above.

### siRNA knockdown of Mfsd2a increased BBB permeability and exacerbated neurological dysfunction after HIBD

RNA interference (RNAi) is a natural process through which target mRNA is degraded, resulting in genetic expression silencing [45]. To screen small interference RNAs (siRNAs) targeting *Mfsd2a* in vitro, we extracted hepatocytes with a high abundance of *Mfsd2a* [46] from WT mice's livers. It was observed that cells presented a spherical shape, with dual or multiple nuclei under the microscope (Fig. S3a). The immunofluorescence staining results of anti-albumin (a hepatocellular marker) showed hepatocytes were successfully obtained (Fig. S3b). We designed three *Mfsd2a*-siRNAs and transfected them separately into hepatocytes in vitro by RNAi. The qRT-PCR analysis revealed that all three *Mfsd2a*-siRNAs downregulated the expression of *Mfsd2a*, among which *Mfsd2a*-siRNA #1 had the highest interference efficiency (Fig. S3c). To determine whether the *Mfsd2a*-siRNA #1 worked in vivo, we injected the siRNA into the brain of WT mice by intra-cerebroventricular (ICV) injection and found it could effectively reduce the Mfsd2a protein expression (Fig. S3d).

**Fig. 6:**
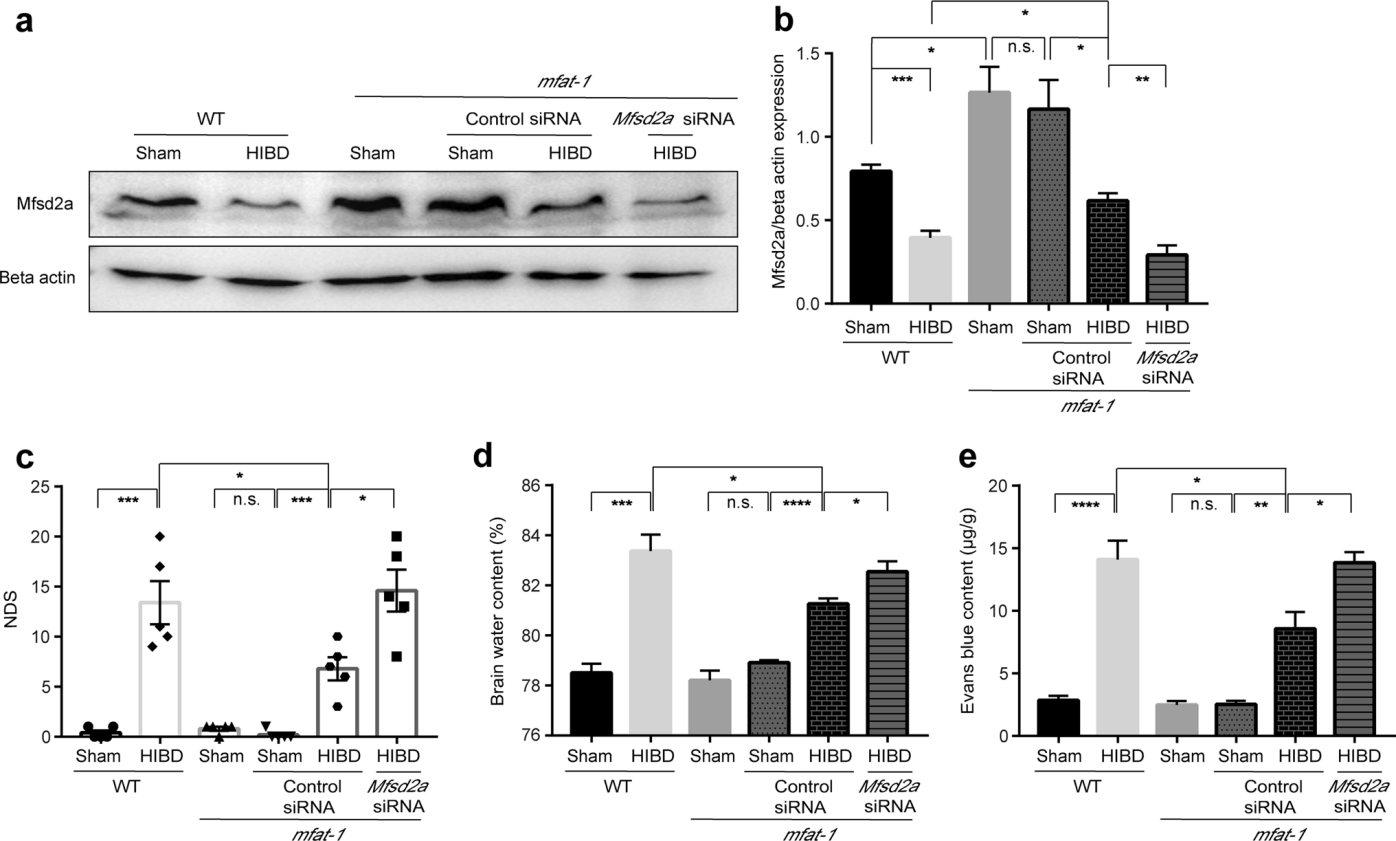
siRNA knockdown of Mfsd2a decreased BBB integrity in *mfat-1* HIBD mice. **a**, **b** Representative western blot images showed the expression of Mfsd2a protein in WT Sham, WT HIBD, *mfat*-*1* Sham, *mfat*-*1* Sham + control siRNA, *mfat*-*1* HIBD + control siRNA, *mfat*-*1* HIBD + *Mfsd2*a-siRNA groups (**a**) and the quantification results (**b**). Specific *Mfsd2a*-siRNA efficiently knocked down Mfsd2a protein in *mfat-1* HIBD mice compared to the control siRNA group (*n* = 4). **c** The NDS results in the six groups (*n* = 5). **d**, **e** The brain water content (**d**) and EB content (**e**) in each group (*n* = 5). *n.s.* no significance; *p < 0.05, **p < 0.01, ***p < 0.001, ***p < 0.0001

To test if *mfat*-*1* mice protected HIBD-induced BBB disruption via elevated Mfsd2a protein expression, we performed knockdown experiments using specific *Mfsd2a*-siRNA by ICV injection. Western blot was used to verify the effect of the manipulation in altering Mfsd2a expression. In contrast to the control siRNA group, siRNA knockdown of *Mfsd2a* significantly reduced the expression of Mfsd2a protein after HIBD in *mfat-1* mice (Fig. 6a, b). Subsequently, we tested the influence of alterations in Mfsd2a expression on BBB permeability by NDS, brain water content, and EB content assays. *Mfsd2a*-siRNA abolished the protective effect against HIBD-induced BBB breakdown in *mfat-1* mice. The results were as follows: the *Mfsd2*a-siRNA group had a higher level of NDS, brain water content, and EB content than the control siRNA in the *mfat-1* group (Fig. 6c–e). Taken together, our experiments showed that the knockdown of *Mfsd2a* aggravated BBB disruption after experimental HIBD, indicating the *mfat-1* mice played a protective role in HIBD-induced BBB disruption correlated with a higher expression of Mfsd2a.

### Inhibition of transcytosis by Mfsd2a in *mfat-1* mice attenuated HIBD-induced BBB damage

**Fig. 7:**
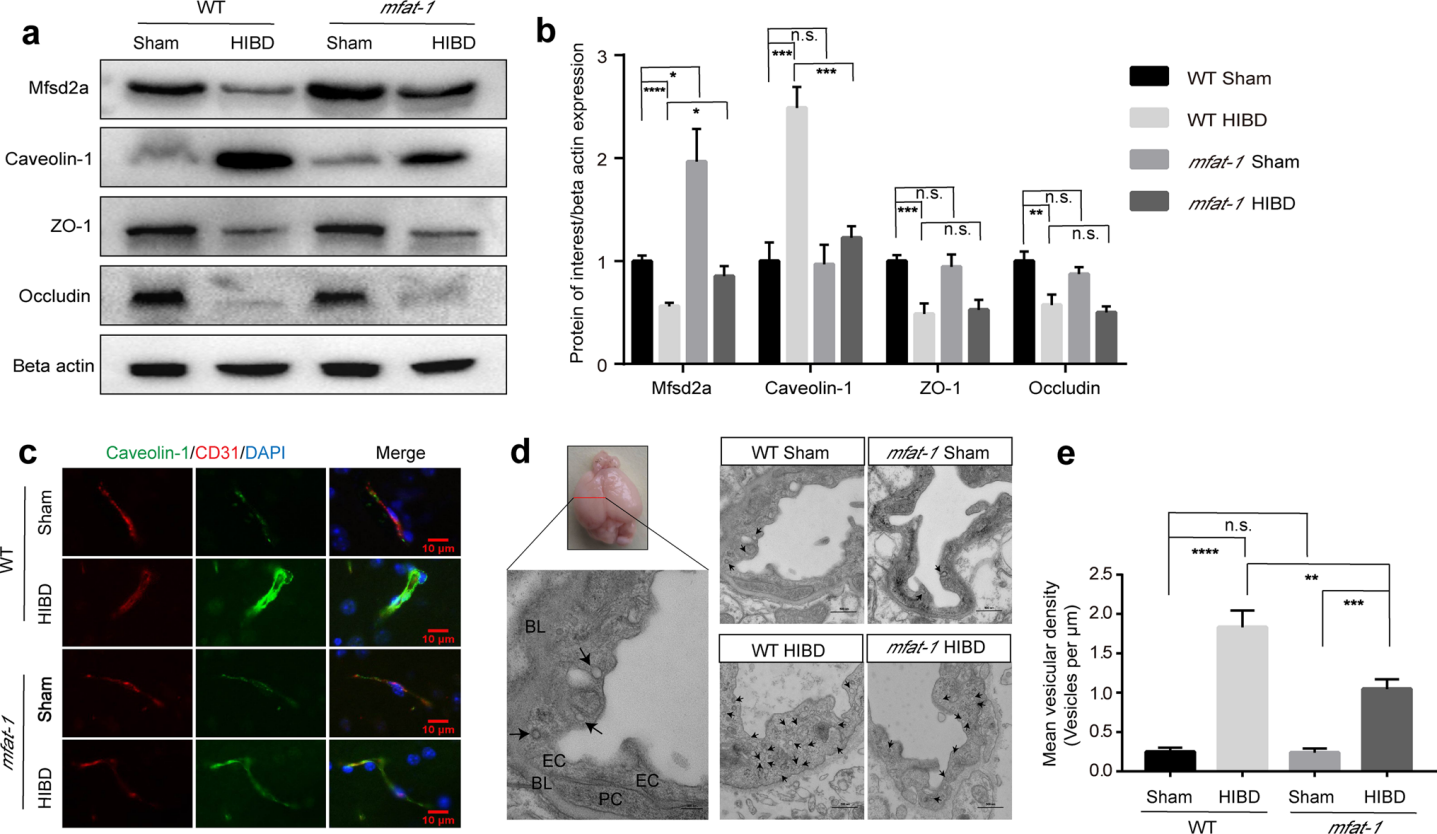
*mfat-1* mice protected HIBD-induced BBB disruption via decreasing vesicle number to inhibit transcytosis.** a**, **b** Changes in Mfsd2a, caveolin-1, ZO-1, occludin protein levels in the WT Sham, WT HIBD, *mfat-*1 Sham, and *mfat-1* HIBD groups measured by western blot (**a**) and corresponding band intensity relative to beta actin (**b**) (n = 7). **c** Anti*-*caveolin*-*1 and anti*-*CD31 double immunofluorescence labeling of cerebral cortex noted the high caveolin*-*1 protein expression in brain vasculature of the WT HIBD group, with a marked reduction in *mfat-1* HIBD mice. Scale bar, 10 μm. **d**, **e** TEM results revealed more vesicles (black arrows) of ECs in mice suffering from HIBD (**d**). Vesicular density quantification of ECs in WT HIBD mice increased compared with *mfat-1* HIBD mice (**e**) (*n* = 3). Scale bars, 200 nm (left); 500 nm (right). *L* lumen, *PC* pericyte, *BL* basal lamina, *n.s.* no significance; *p < 0.05, **p < 0.01, ***p < 0.001, ***p < 0.0001

Given the observed results showing the protection of BBB in *mfat-1* mice was related to the expression level of Mfsd2a after HIBD, we next sought to explore the underlying mechanism by which Mfsd2a affects the BBB permeability. Consistent with the results of Fig. 6a, b above, due to the increased expression of Mfsd2a protein in the brain of *mfat*-*1* mice, the content of Mfsd2a in *mfat*-*1* mice after HIBD was significantly higher than that of WT HIBD animals (Fig. 7a, b). As expected, there was no significant difference in the expression level of ZO-1 and occludin after HIBD, regardless of whether the mice were *mfat*-*1* or WT (Fig. 7a, b). This result suggested the BBB integrity in *mfat*-*1* mice was related to Mfsd2a rather than TJ-related proteins.

Substance transport from the blood to the brain parenchyma mainly depends on transcellular transport, and caveolin-1 (caveolae marker) is crucial for transcytosis in ECs [47, 48]. Therefore, we examined the expression level of caveolin-1, which yielded an essentially opposite trend as those of the Mfsd2a protein detected above (Fig. 7a–c). Next, the ultrastructure of the BBB was observed in the damaged brain tissues from coronal sections of the brain to further observe the changes in micromorphology after HIBD using transmission electron microscopy (TEM) (Fig. 7d). Compared to WT Sham mice, WT HIBD mice had more caveolae-like vesicles, which were partly responsible for peripheral endothelial permeability (Fig. 7d, e). Significantly, the *mfat-1* HIBD mice have fewer vesicles in the BBB (Fig. 7d, e). *mfat-1* animals could partially offset pericyte loss after HIBD, which is typically linked with increased transcytosis (Fig. S4a, b) [49]. Collectively, these findings revealed that elevated Mfsd2a is essential for maintaining BBB physiological permeability post-HIBD in *mfat-1* mice, and its protective effect on the BBB was achieved by inhibiting transcytosis.

## Discussion

BBB disruption is a critical pathological manifestation of HIBD, contributing to additional parenchymal damage and edema. Therefore, exploiting means to protect the BBB is a promising disease-control strategy for treatment in the early period after HIBD. In our study, we first discovered that the neuronal damage was reduced and the physiological permeability of the BBB was partly preserved in the *mfat*-*1* transgenic mice following HIBD. We further proved this was associated with elevated Mfsd2a expression on the BBB, resulting from more LPC-DHA entering the brain through blood circulation. Furthermore, we demonstrated that the *mfat-1* mice could significantly mitigate the HIBD-induced BBB disruption by modulating vesicular transcytosis across the cerebral endothelium.

BBB breakdown and dysfunction lead to leakages of harmful blood components into the CNS in disease states, contributing to neurological deficits and aggravating disease [50]. One of the main features of BBB disruption is cerebral edema, and the AQP4 expression is decreased in the case of BBB disruption (such as vasogenic cerebral edema) [33]. The result of AQP4 indirectly demonstrated that the BBB was compromised and the permeability was increased after HIBD, during which multiple mechanisms were implicated in forming cerebral edema. Pericyte loss, one of the hallmarks of BBB dysfunction [51], could be partially reversed in the *mfat-1* mice (Fig. S4a, b). We also found that *mfat-1* mice mitigated the loss of neurons after HIBD and reduced the number of activated astrocytes in our study (Fig. S5a, b). However, the underlying mechanism awaits further investigation.

Plasma DHA exists in several molecular forms. Among them, only the albumin-bound forms (free (unesterified) DHA and LPC-DHA) [52, 53] are known to be transported through the BBB. Previous studies have shown that free DHA is the predominant source of DHA in the brain, but more recent studies supported the notion that LPC is the major carrier of DHA responsible for brain enrichment [54–56]. For example, dietary LPC-DHA enriches brain DHA efficiently, while dietary free-DHA at an equivalent dose has little effect on brain DHA content [54]. Our study found that LPC-DHA content increased in the brains of WT mice after long-term feeding with an LPC-DHA-rich diet, providing evidence for the above point of view, that LPC-DHA can effectively enrich DHA in the brain. Mfsd2a protein expression was found to be significantly higher on the BBB of the brain in *mfat-1* mice, which was related to the transport of higher levels of LPC-DHA in the plasma into the brain, as Mfsd2a could transport DHA into the brain in the form of LPC but not unesterified fatty acids. Furthermore, increasing the concentration of LPC-DHA effectively increased Mfsd2a expression on the BBB in WT mice. The mechanism by which LPC-DHA manipulates Mfsd2a is unknown, and it is unclear whether this process is solely determined by LPC-DHA. In the future, we will focus on analyzing the specific molecular forms of DHA and their corresponding contents in the plasma and brain of the *mfat-1* transgenic mice.

In this study, we clarified that Mfsd2a-dependent regulation of transcytosis on the BBB was primarily through the caveola-mediated pathway. The barriers of CNS require specialized characteristics to maintain its integrity, including TJs and typically low rates of transcytosis. Transcytosis pathways in the ECs can be categorized into clathrin-mediated, caveola-mediated, and other pathways. In our study, the size of the vesicles observed in TEM images fell into the diameter range of caveolae. WT and *mfat-1* mice subjected to HIBD showed no statistical difference in TJ-related proteins, which provided evidence that the protective roles of Mfsd2a in BBB may be independent of TJ after HIBD. Considering that Mfsd2a could serve as a therapeutic target for regulating transcytotic mechanisms in CNS ECs, our study may shed light on the development of new therapeutic strategies in BBB-related brain diseases. Furthermore, transcytosis often serves as a crucial route for drug delivery in the CNS. While maintaining an immune-exempt environment, the barriers in the brain sometimes also become obstacles for drug delivery to desired sites when treating neuronal diseases. Better ways to modulate transcytosis may assist in the selective delivery of medicine to the CNS tissues.

Nowadays, the following questions still require follow-up research: (i) why does the brain selectively enrich plasma-derived DHA? (ii) what is the specific interaction mechanism between Mfsd2a and caveolin-1? (iii) Mfsd2a transports LPC-DHA through a direct or indirect mechanism? (iv) do different concentrations of DHA in the brain regulate the transport of LPC-DHA by Mfsd2a protein? (v) the molecular mechanism of LPC-DHA transport by Mfsd2a remains unknown. (vi) Mfsd2a protein expression decreased sharply after HIBD but gradually recovered over time, and the specific mechanism was unclear. For this final question, although we found that Mfsd2a protein expression in the brain was close to that of the Sham group one week after HIBD (Fig. 5a–d), the direct and indirect brain damage caused by HIBD-induced BBB disruption during this period was irreversible.

Based on this study, our understanding of the *mfat*-*1* gene acting on the BBB after HIBD at the molecular and cellular level will continue to grow in rodent models. The question remains to what extent these findings are translatable to the human BBB. Dietary supplementation with a specific form of DHA is a novel treatment for reducing the risk of neurodegenerative disorders [57]. For people with high-risk factors for HIBD, daily intake of LPC-DHA may be an effective prevention and treatment strategy for HIBD-induced BBB disruption to protect the brain against HI as preservation of the BBB is a common goal among neuroprotective therapies.

In conclusion, our data support that *mfat-1* transgenic mice have higher expression of Mfsd2a on the BBB, which partly sustains BBB permeability via vesicular transcytosis rather than the TJ-related protein to ameliorate HIBD. Therefore, daily intake of an appropriate dose of LPC-DHA could be potential prevention and treatment for poor outcomes caused by HIBD. In addition, findings from this study can further our understanding of basic transcytosis regulation and may provide a feasible direction for treating BBB disruption-related diseases and developing drug delivery systems.

## Materials and methods

### Animals and housing

All animal husbandry and experiments were carried out in accordance with the requirements approved by the Institutional Animal Care and Use Committee (IACUC 2012032) of Nanjing Medical University. *mfat*-*1* transgenic mice were obtained via prokaryotic microinjection as previously described [58]. An F0 mouse (*mfat-1* ^Tg/+^) (Fig. S6a, b) mated with C57BL/6 J mice to generate *mfat*-*1* heterozygous mice (*mfat-1* ^Tg/+^) and WT (*mfat-1* ^+/+^) littermates. From beginning to end, the *mfat-1* ^Tg/+^ mice mated with littermates of *mfat-1* ^+/+^ mice to maintain a single number of the *mfat-1* gene in transgenic offspring. Male mice (6–8 weeks old) were selected in the present study to exclude gender interference. All animals were born and bred in a specific pathogen-free facility, housed on a 12-h light/dark cycle, and provided ad libitum access to food and water.

### HIBD mouse experimental model and NDS determination

To better understand the whole study, a timetable graphic outline of the experimental design was presented in Fig. S7. The male mice aged between 6 and 8 weeks were used to establish the HIBD experimental model as previously reported [23]. In brief, the mice were positioned supine in a surgical stereomicroscope (Nikon) under induction and maintenance of 1–2 vol.% isoflurane (RWD Life Science) anesthesia. The unilateral common carotid artery (CCA) (left) was carefully isolated and permanently ligated with 7–0 silk thread. After a 2 h recovery in a normal environment, the experimental mice were placed in a chamber with nitrogen-filled air to maintain an oxygen concentration of 8% for 50 min. Sham-operated mice were anesthetized by leaving the left CCA unligated.

To examine the NDS, including circular behavior, climbing, and so on, a 28-point neurological impairment scale was used [59]. The scoring was carried out by two researchers unaware of the animal classifications. The final score for each mouse was calculated by averaging the above two scores.

### Tissue preparation and histological examination

Animals were euthanized one day post-HIBD. The choice of this time point was based on the experimental results showing that the lowest expression of Mfsd2a protein in the brain was observed the day post-HIBD (Fig. 5a–d). Thus, this time point was chosen to carry out all other experimental analyses. The brain of mice was dissected immediately after transcardial perfusion with pre-cooled PBS and then fixed in 4% paraformaldehyde (PFA/PBS) over 24 h at room temperature (RT). Subsequently, the brain tissues were dehydrated and embedded in paraffin. The 5 μm sections of the coronal plane were performed with H&E, and Nissl's staining followed standard protocol [60] to analyze HIBD-induced neuropathological damages. Images were captured using a microscope (Nikon).

### Infarct volume measurement

At 24 h post-HIBD, the brain of mice was harvested rapidly after cervical dislocation and placed at −40℃ for 10 min to harden the tissue. The brains were sliced into two mm-thick coronal sections to be incubated with 2% TTC (Sigma-Aldrich) at 37℃ for 30 min, followed by 4% PFA. The infarct volume (stained white) was measured by ImageJ software.

### MRI analysis

Lesion area was determined from proton density, and T2WI was obtained with a standard spin-echo pulse sequence on a Biospec 7T/20 USR MRI system (Burker). Mice were scanned under isoflurane anesthesia while in ventral recumbency with the head on a circularly polarized extremity coil post-HIBD. All mice's scans ranged from the top to the base of the skull. The scanning parameters were as follows: RARE sequence T2WI: TR, 3000 ms; TE, 38 ms; FA, 90 deg; SI, 0.8 × 0.8 mm; FOV, 22 × 20 mm; layer thickness, 0.8 mm; and layer gap, 0.8 mm.

### qRT-PCR

Total RNA of the affected cerebral hemisphere was extracted by TRIzol reagent (Invitrogen) and reverse transcribed into cDNA using the HiScript®II QRT SuperMix (Vazyme) according to the manufacturer's instructions. The expression of several housekeeping genes has been shown to vary due to tissues or cell types [61]. A single housekeeping gene may not meet the criteria of an ideal reference gene. NormFinder, a statistical algorithm, evaluates each single reference's expression stability and considers intra- and intergroup variations for normalization [61]. In both the brain tissues and hepatocytes of WT mice, we found the *beta-actin* gene was the most stable by NormFinder analysis compared with commonly used reference genes of *beta tubulin* (forward: 5´-GAGGGTGAAGATGAGGCTTG-3´ and reverse: 5´-GAAAGACCATGCTGGAGGAC-3´), *GAPDH* (forward: 5´-TGTGTCCGTCGTGGATCTGA-3´ and reverse: 5´-TTGCTGTTGAAGTCGCAGGAG-3´) and *HPRT1* (forward: 5´-CGTCGTGATTAGCGATGATG-3´ and reverse: 5'-TCCAAATCCTCGGCATAATG-3') (Fig. S8a, b). The target genes were amplified with the SYBR Green PCR Master Mix (Abclonal) in three replicates. Cycling parameters were hot starting at 95℃ for 3 min, followed by 40 cycles of amplification at 95℃ for 5 s and 60℃ for 30 s. The primers specific for *Mfsd2a*, *ZO*-*1,* and *occludin* genes of mice were as follows: *Mfsd2a* forward: 5'-AGAAGCAGCAACTGTCCATTT-3' and reverse: 5´-CTCGGCCCACAAAAAGGATAAT-3´; *ZO*-*1* forward: 5´-ACTCCCACTTCCCCAAAAAC-3´ and reverse: 5´-CCACAGCTGAAGGACTCACA-3´; *Occludin* forward: 5´-CCTTCTGCTTCATCGCTTCCTTA-3´ and reverse: 5´-CGTCGGGTTCACTCCCATTAT-3´. *Beta actin* (forward: 5´-AGGGCTATGCTCTCCCTCAC-3´ and reverse: 5´-CTCTCAGCTGTGGTGGTGAA-3´) was used as an internal control. We used the melting curve analysis to evaluate the amplification specificity and the 2^−ΔΔCt^ method to measure the relative mRNA expression levels.

### Western blot analysis

The western blot was performed as described previously [62]. Proteins (20–30 μg) of the left cerebral hemisphere extracted were separated by SDS-PAGE gel electrophoresis (Proteinbio) and transferred onto polyvinylidene fluoride (PVDF) membranes (Bio-rad) by electroblotting. The membranes were blocked by 5% skim milk dissolved with Tris-buffer saline (TBS, containing 0.1% Tween-20) for 1 h at RT and then incubated with primary antibody at 4℃ overnight. These primary antibodies were used: anti-Mfsd2a (Thermo Fisher Scientific), anti-ZO-1 (Abclonal), anti-occludin (Abcam), anti-AQP4 (Abclonal), anti-albumin (Abclonal), anti-caveolin-1 (CST), anti-beta actin (Abcam). Horseradish peroxidase (HRP)-conjugated secondary antibody was incubated with the membranes at RT for 1 h. The secondary antibody was the anti-rabbit antibody (Abcam). The proteins were detected by the ECL chemiluminescence system (Vazyme), and the corresponding images were captured using a ChemiDoc Touch Imaging System (Bio-Rad). The band intensity was analyzed by ImageJ software.

### Morris water maze test

The Morris water maze was performed as described previously [63]. Briefly, mice were trained in the Morris water maze three times a day for 5 days, and data were recorded from day 2. The water tank with a depth of 50 cm and diameter of 180 cm was filled with water to a height of 30 cm. The water was controlled at 20–22℃ and stained with non-toxic dyes. The platform was placed 2 cm below the surface of the water, which color is as different as possible from the mouse coat color to capture by the machine accurately. The starting point changed every day. Each test continued until either the mice found the platform or until 60 s had elapsed. The mice were placed on the platform for 20 s after each test. The swimming distance and escape latency were recorded and analyzed.

### TUNEL staining

TUNEL staining was used to detect DNA fragmentation and apoptotic bodies in the cells. In short, after routine dewaxing and rehydration, the paraffin coronal sections were incubated with proteinase K (20 mg/mL) at 37℃ for 25 min, followed by the TUNEL reaction mixture (Roche) according to instructions, maintained in a 37℃ incubator for 1 h. Fluorescent signals were examined using a fluorescence microscope (Nikon) and quantification analysis.

### Immunofluorescence staining

For immunofluorescence staining, OCT-embedded frozen section (tissue) or cell climbing side (cell) was fixed with pre-cooled acetone for 10 min at RT, followed by blocking with goat serum for 1 h. Samples were incubated with primary antibodies at 4℃ overnight. These primary antibodies were used: anti-NeuN (Millipore-Chemicon), anti-CD31 (RD Systems), anti-caveolin-1, anti-glial fibrillary acidic protein (GFAP) (Abcam), anti-desmin (Abclonal), anti-cleaved caspase 3 (CST) and anti-albumin. After washing in PBS three times, the corresponding secondary antibody was subsequently used in the sample at RT for 1 h. The secondary antibodies included Alexa Fluor 594 (goat anti-rabbit) (Abcam), Alexa Fluor 488 (Goat anti-mouse) (Abcam), and Alexa Fluor 488 (Goat anti-chicken) (Invitrogen). Finally, the sample was washed and stained with diaminobenzidine/imidazole (Sigma). Immunofluorescence images were acquired by a fluorescence microscope (Nikon).

### BBB permeability assays

A 2.5% solution of EB dissolved in normal saline was administered to mice by tail vein injection (4 mL/kg of body weight) and was allowed to circulate for three hours. Afterward, the mice were anesthetized and transcardially perfused with ice-cold PBS to clean any remaining EB in the blood vessels. The brain tissues were excised and homogenized in formamide solution (3 mL/100 mg of brain tissue), incubated at 60℃ water bath for 24 h. EB content was measured by a spectrophotometer (BioTek) at 630 nm and calculated according to a standard curve normalized to tissue weight (μg of EB/g of tissue).

The permeability assay using a FITC-conjugated tracer (dextran, 40 kDa) (Sigma-Aldrich) was performed as previously described in vivo [64]. To assess BBB permeability, mice were injected with dextran at a concentration of 10 mg/mL through the tail vein. After 10 min, animals were killed by cervical dislocation and then isolated brains. The brains were fixed in 4% PFA and soaked in sucrose until sinking to the bottom. Ten micrometer coronal slices were obtained with a freezing microtome (Leica) and stained with blood vessel marker anti-CD31 antibody. Fluorescent signals were examined using a fluorescence microscope (Nikon).

### Brain water content

The brain of the mice was removed and weighed immediately for wet weight. After being dried in an oven at 100℃ for 24 h, the brain was weighed as dry weight. The percentage of brain water content was calculated based on the following formula: [(wet weight − dry weight)/wet weight] × 100%.

### LPC-DHA-rich diet preparation

In Antarctic krill oil, the main component of ω-3 PUFAs is DHA and eicosapentaenoic acid (EPA), existing in combination with phospholipids (mostly located in position 2) [65]. Here we prepared LPC-DHA-rich diet as described by Hu et al. previously. In brief, 10 g of krill oil (NYO3) was mixed with 500 mL Tris–HCl buffer (PH 7.0) and added 100 μL phospholipase A1 (PLA1) from Aspergillus oryzae (≥ 10 KLU/g) (Aladdin) to catalyze the hydrolysis of an acyl group from position 1 of lecithin to yield lysolecithin (Fig. S9a). The reaction mixture was incubated at 50℃ along with shaking at 180 rpm for 1 h, inactivated the PLA1 at 90℃, removed water by rotary evaporator, the lipids were extracted finally. Lipid extract of PLA1-treated krill oil was analyzed by thin-layer chromatography (TLC) (Fig. S9b) using trichloromethane, methanol, and ammonium hydroxide (65:30:4, V/V/V) as the developing solvent, and analyzed by LC–MS/MS (Shimadzu Corporation) (Fig. S9c).

Yalagala et al. have demonstrated that molecular forms of dietary DHA except LPC are ineffective at enriching brain DHA level, where 3.6 μmol of LPC per 3 g of diet [55]. Through calculation, the LPC-DHA-rich diet contained 10 g of PLA1-treated krill oil and 57 g of corn oil per kg to give 7% total fat in our study. Oils were blended with the rodent basic diet, pelleted, and vacuum sealed by Xietong Organism.

Male C57BL/6 J mice (age, 8 weeks) were purchased from the Laboratory Animal Center of Nanjing Medical University, randomly divided into two groups, and fed regular chow diet (Xietong Organism) or LPC-DHA-rich diet separately ad libitum for three months. We harvested the brain of mice under deep anesthesia. All samples were flash-frozen in liquid nitrogen and stored at –80℃ until analysis.

### LC–MS/MS analysis

LC–MS/MS can accurately quantify fatty acids. Total lipids from the mouse's brain were extracted according to the chloroform/methanol/water method described previously [66]. LC–MS/MS conditions were as follows: injection volume: 2 μL; flow rate: 0.35 mL/min; mobile phase A: acetonitrile–water (60:40, V/V); mobile phase B: acetonitrile–isopropanol (10:90, V/V); curtain gas: 35 psi; positive ion spray voltage: 4500 V; negative ion spray voltage: −4500 V; temperature: 450℃; column temperature: 45℃; gas 1: 55 psi; gas 2: 55 psi.

### Isolation and culture of primary mouse hepatocytes

After anesthesia, the WT healthy experimental mice (male, 8–10 weeks old) were secured limbs using needles and made a ‘‘U’’-shaped incision on the abdominal cavity to fully expose the portal vein and vena cava. The portal vein was cannulated at a flat angle relative to the vein to perfuse with preheated sterile D-Hank's solution (37℃) by a peristaltic pump, aiming to wash out the blood and circulating cells from the liver. Upon the appearance of white spots forming in the liver, immediately cut the inferior vena cava with scissors. After the liver was turned yellow-white, the perfusate was replaced by a digestion solution (D-Hank's solution containing 0.1 mg/mL IV collagenase (Sigma-Aldrich) to digest the liver. During digestion, clamping the inferior vena cava for several seconds with forceps could greatly facilitate efficient digestion. Finally, the liver was dissected out gently followed by removing the gall bladder, and the hepatocytes were released into suspension and cultured in Dulbecco's modified eagle medium (DMEM) medium supplemented with 10% fetal bovine serum.

### Transfection of siRNA into the brain

Specific siRNAs against mouse *Mfsd2a* (ID: 76574) were provided by Ribobio via the online tool (https://rnaidesigner.thermofisher.com/rnaiexpress/sort.do). The following three different *Mfsd2a*-targeted sequences were used in our study: siRNA-1 (5´-GGATGTGGCTAAGGTGGAA-3´), siRNA-2 (5´-GCACCGAGCCCATATTCTT-3´), and siRNA-3 (5´-CCTGAAGCCACCACACAAA-3'). All three of these siRNAs were used separately to knock down the expression of *Mfsd2a* to test their interference efficiencies in vitro in hepatocytes (Fig. S3a). siRNA-1, the most efficient of the three siRNAs (Fig. S3c), was used for the experiments in our study. According to the manufacturer's instructions, 5 nM of *Mfsd2a*-siRNA powder and 5 nM of control-siRNA powder was dissolved in 10 μL RNase-free water, respectively, and then given by ICV injection at 48 h before operation.

After anesthesia, animals were placed in a stereotactic frame (WPI) and performed ICV injection as previously described [67]. According to the atlas, over the left cerebral hemisphere, 1.13 mm lateral to the left of the midline and 0.59 mm posterior to the bregma, a burr hole was drilled through the skull. A 10 μL Hamilton syringe needle was inserted into the left lateral ventricle, controlling the depth at 3.8 mm below the skull surface. siRNA solution was injected into the left ventricle at the rate of 0.5 μL/min. The needle was withdrawn after holding the original location for an additional 10 min in case of liquid leakage. To verify that the injection was carried out properly and stably, we also used mice subjected to ICV injection of the same volume of Giemsa dye (Sigma-Aldrich). As shown in Fig. S3f, the distribution of the injected dye throughout both sides of the lateral ventricles was confirmed.

### TEM

After perfusion, the one mm^3^ fragments of the mouse cerebral cortex were fixed in 4% glutaraldehyde at 4℃ overnight, followed by dehydration and embedding. Imaging was performed on 80 nm ultra-thin sections counterstained with uranyl acetate and lead citrate by a JEM-1010 transmission electron microscope (JEOL). Random microvessel areas from the brain tissue of each mouse were oriented for vesicular density quantification analysis.

### Southern blot analysis

Southern blot is a hybridization-based technique that can effectively identify transgenic insert/copy numbers [68]. The total genomic DNA (10 μg) was extracted according to the manufacturer's instructions (QIAGEN) and digested by the *Bbs*I restriction enzyme (NEB). Genomic DNA fragments were transferred to a membrane, and incubating specific probe targeting *mfat-1* gene amplificated with digoxigenin (DIG)-dUTP (Roche) at 65℃ over 16 h in a hybridization oven (primers used for probe amplification, forward: 5´-GGACCTGGTGAAGAGCATCCG-3´; reverse: 5´-GCCGTCGCAGAAGCCAAAC-3´). Then the membrane was incubated with an anti-DIG antibody conjugated with alkaline phosphatase (AP) (Roche) for 1 h at RT. The *mfat-1* gene was detected by the CDP star (Roche), and the corresponding image was captured using a ChemiDoc Touch Imaging System (Bio-Rad).

### Statistical analysis

All quantitative data were presented as mean ± standard error of the mean (SEM). Data for two groups were analyzed by a two-tailed unpaired Student's *t* test. SEM was calculated for all quantitative data and displayed as error bars in graphs. The survival/death rates for the two groups were analyzed by the Chi-square test. A *p* value of 0.05 was set as the threshold value of statistical significance.

### Supplementary Information

Below is the link to the electronic supplementary material.

Supplementary file1 (DOCX 17227 KB)

## Data Availability

The data sets generated during and/or analyzed during the current study are available from the corresponding author upon reasonable request.
