# Supplementary material for: Mfsd2a attenuated hypoxic-ischemic brain damage via protection of the blood–brain barrier in mfat-1 transgenic mice
Source: Cell Mol Life Sci. 2023 Feb 23;80(3):71. doi: 10.1007/s00018-023-04716-9 (PMC9950179; doi:10.1007/s00018-023-04716-9)
Supplement: Supplementary file 1 — Supplementary file1 (DOCX 17227 KB) [file 18_2023_4716_MOESM1_ESM.docx]

**Supplementary data**


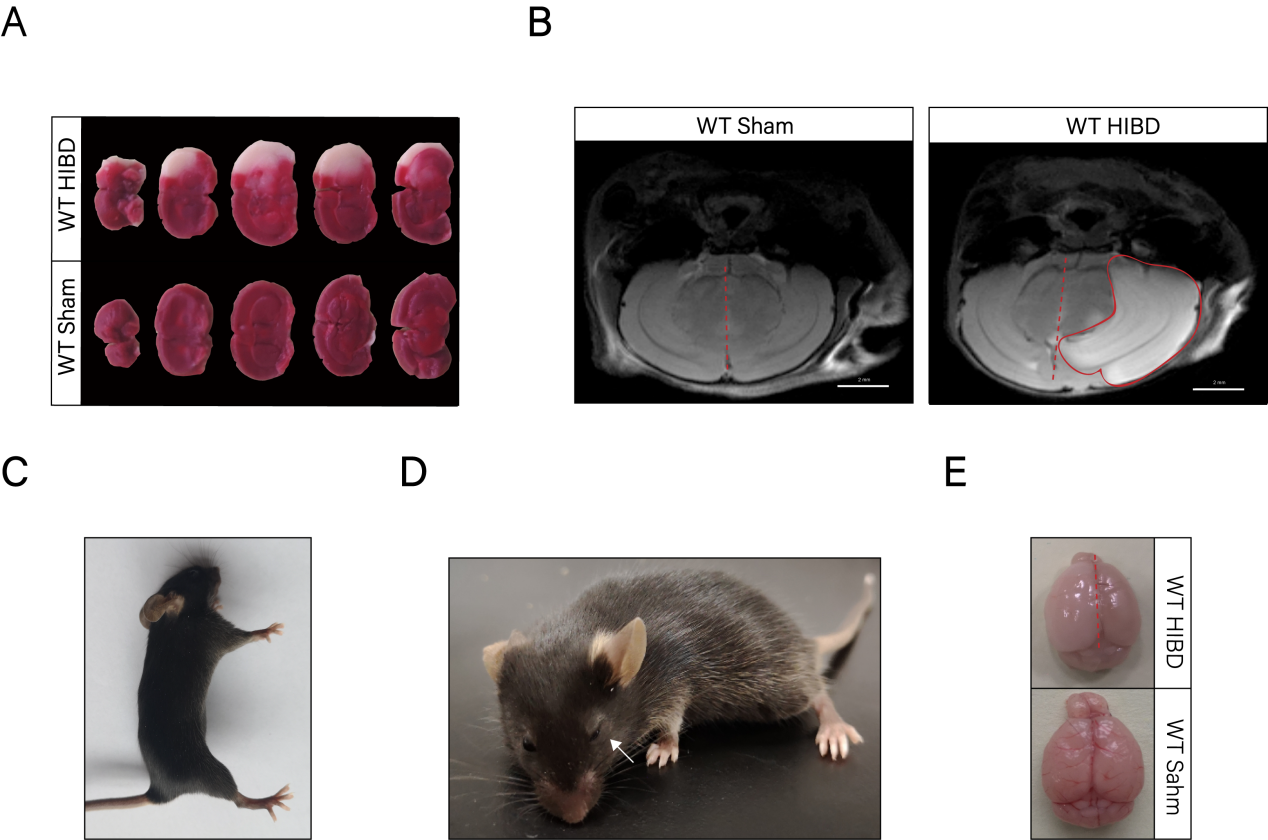


**Fig. S1** Symptoms of mouse HIBD model. **a** Representative images of TTC-stained brain sections in the two groups. The normal brain tissue appeared red, with infarct loci stained white. **b** Massive cerebral infarction in the left cortical region after HIBD in T2WI of MRI. The affected cerebral hemisphere occurred cerebral edema, and the brain midline shifted. The solid red lines indicate the cortical ischemic infarction area. **c** The body of the WT HIBD mouse leaned to the affected side (left). **d** HIBD mice could barely open their eyes on the left side. The white arrow indicates the eye of the affected side. **e** Overall view of the whole brain of WT Sham and WT HIBD mice. The left cerebral hemisphere of the WT HIBD mouse appeared to have apparent ischemic infraction together with edema. The dotted red line represents the brain midline. Scale bar, 2 mm


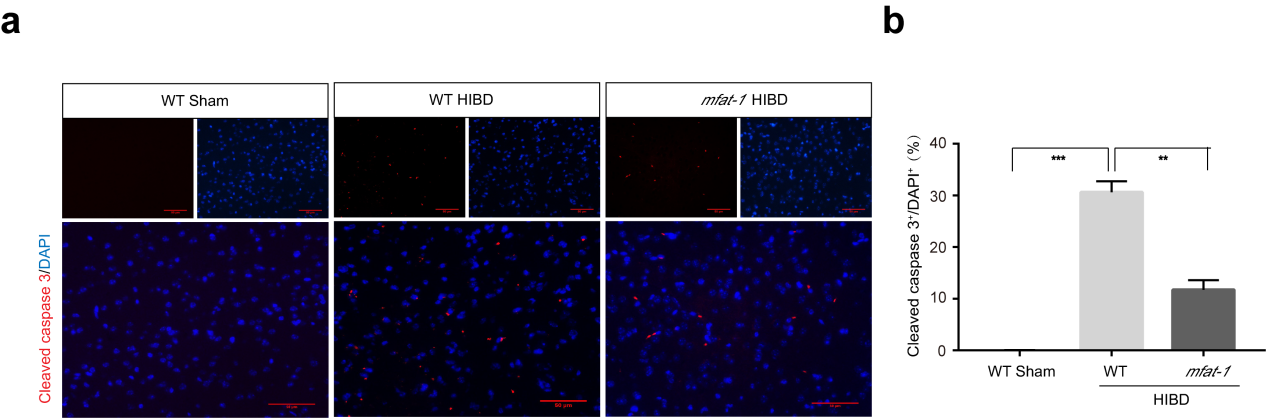


**Fig. S2** *mfat-1* mice decreased the level of apoptosis in the brain post-HIBD. **a** Representative immunohistochemistry images of cleaved caspase 3 together with DAPI. Scale bar, 50 μm. **b** The proportion of cleaved caspase 3 positive cells among DAPI positive cells was counted (n = 3). ***p*<0.01, ****p*<0.001


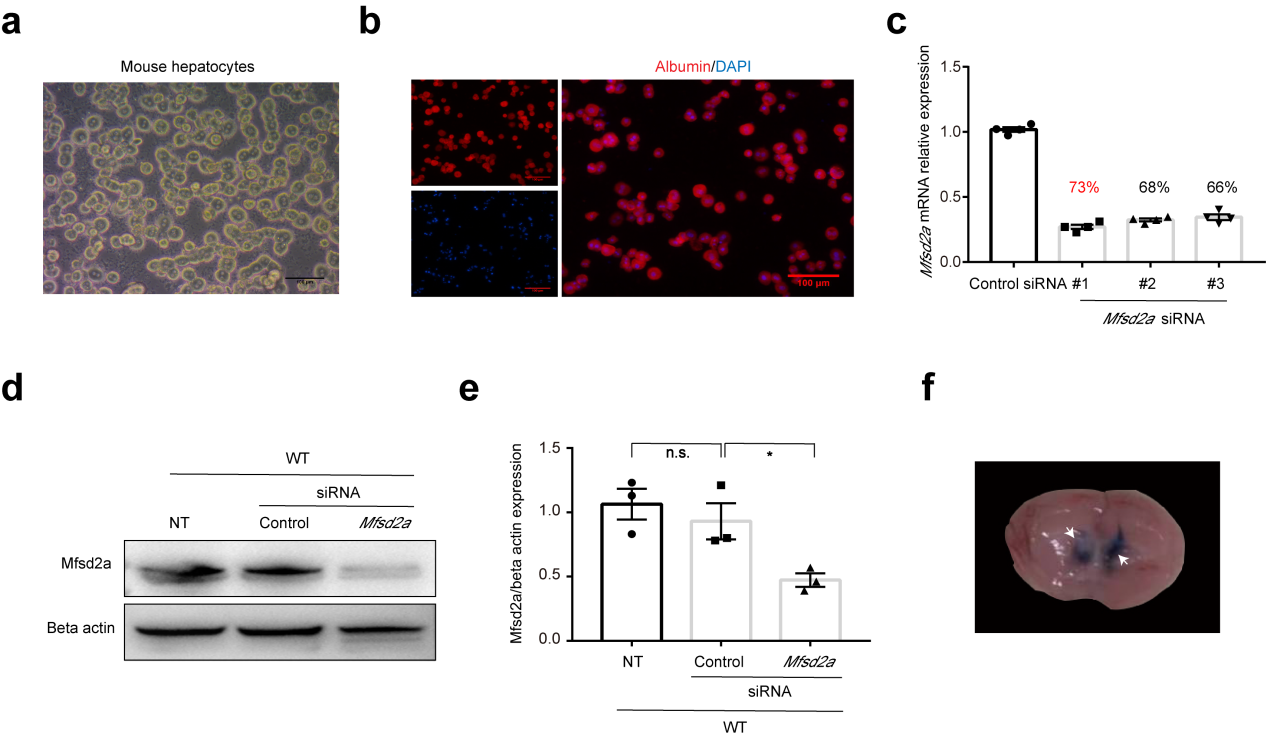


**Fig. S3** The screening of *Mfsd2a*-siRNAs used mouse hepatocytes in vitro and ICV injection. **a** Mouse hepatocytes under the microscope. **b** Anti-albumin (red) immunofluorescence staining indicated no mixing with other cells in mouse hepatocytes. **c** The efficiency of three siRNAs knocked down *Mfsd2a* mRNA in hepatocytes in vitro by RT-qPCR, compared with control-siRNA. Among them, siRNA #1 had the highest interference efficiency (n = 4). **d and e** Western bolt showed that the expression of Mfsd2a protein was significantly decreased in mice injected with *Mfsd2a*-siRNA #1 by ICV compared with mice injected with control siRNA (**d**) and quantitative results (**e**) (n = 3). **f** After the mouse was injected with Giemsa dye by ICV injection, the dye was obviously distributed in the left and right lateral ventricles, proving the accuracy and stability of ICV injection in our manipulations. The white arrows represent the dye distributed in the two lateral ventricles. Scale bar, 100 μm. **p*<0.05


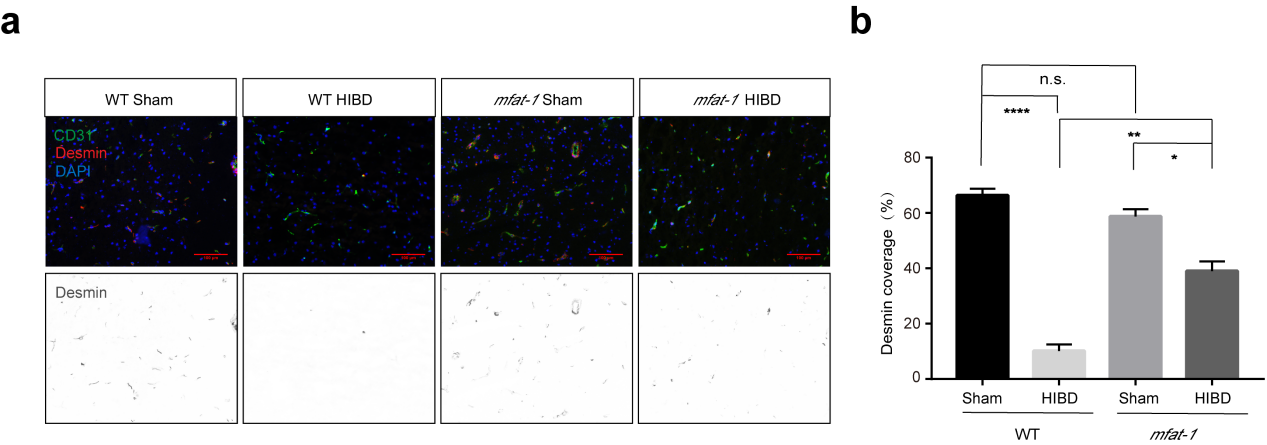


**Fig. S4** *mfat-1* mice alleviated pericyte loss after HIBD. **a** Representative photomicrographs of immunohistochemistry for desmin (pericyte marker), CD31, together with DAPI. Scale bar, 100 μm. **b** The CD31-based coverage of desmin indicated that *mfat-1* mice attenuated pericyte loss after HIBD (n = 3). n.s., no significance; **p*<0.05, ***p*<0.01, *****p*<0.0001


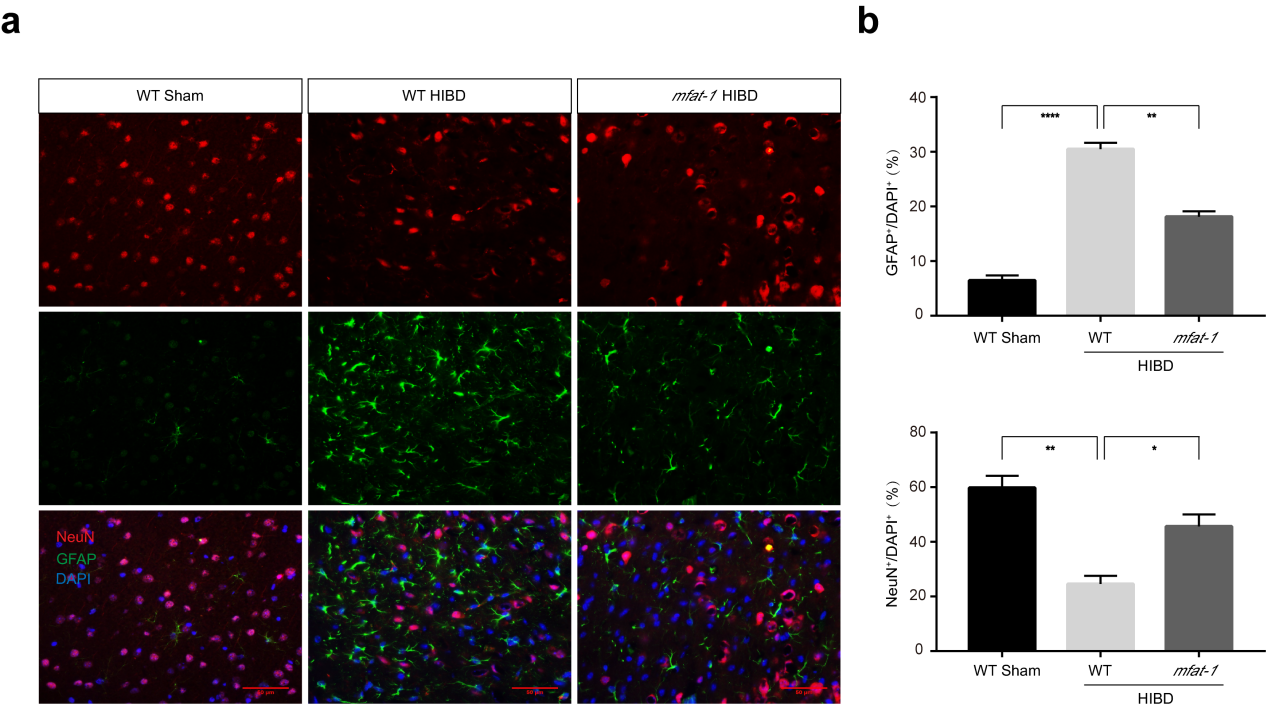


**Fig. S5** *mfat-1* mice attenuated neuronal damage and astrocyte activation post-HIBD. **a** Representative photomicrographs of immunohistochemistry for anti-GFAP antibody and anti-NeuN antibody as well as nuclear acid staining with DAPI in the cortex of the WT Sham, WT HIBD, and *mfat-1* HIBD groups. Scale bar, 50 μm. **b** The proportion of GFAP-positive cells among DAPI-positive cells and NeuN-positive cells among DAPI positive cells were counted (n = 3). **p*<0.05, ***p*<0.01, *****p*<0.0001


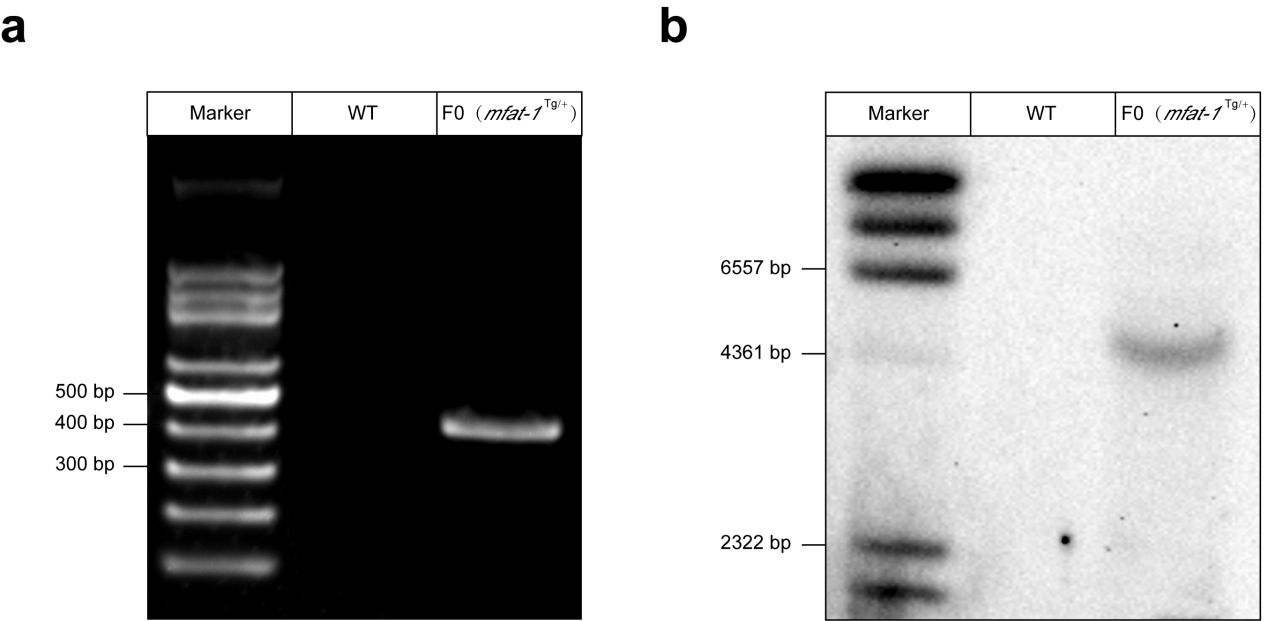


**Fig. S6** The genotype of the F0 mouse was *mfat-1*^Tg/+^. **a** Gel electrophoresis result of PCR products that amplified the *mfat-1* gene in WT and F0 mice. The amplified product size of the F0 mouse was 438 bp. **b** Southern blot detected the copy number of the *mfat-1* gene in the F0 mouse.


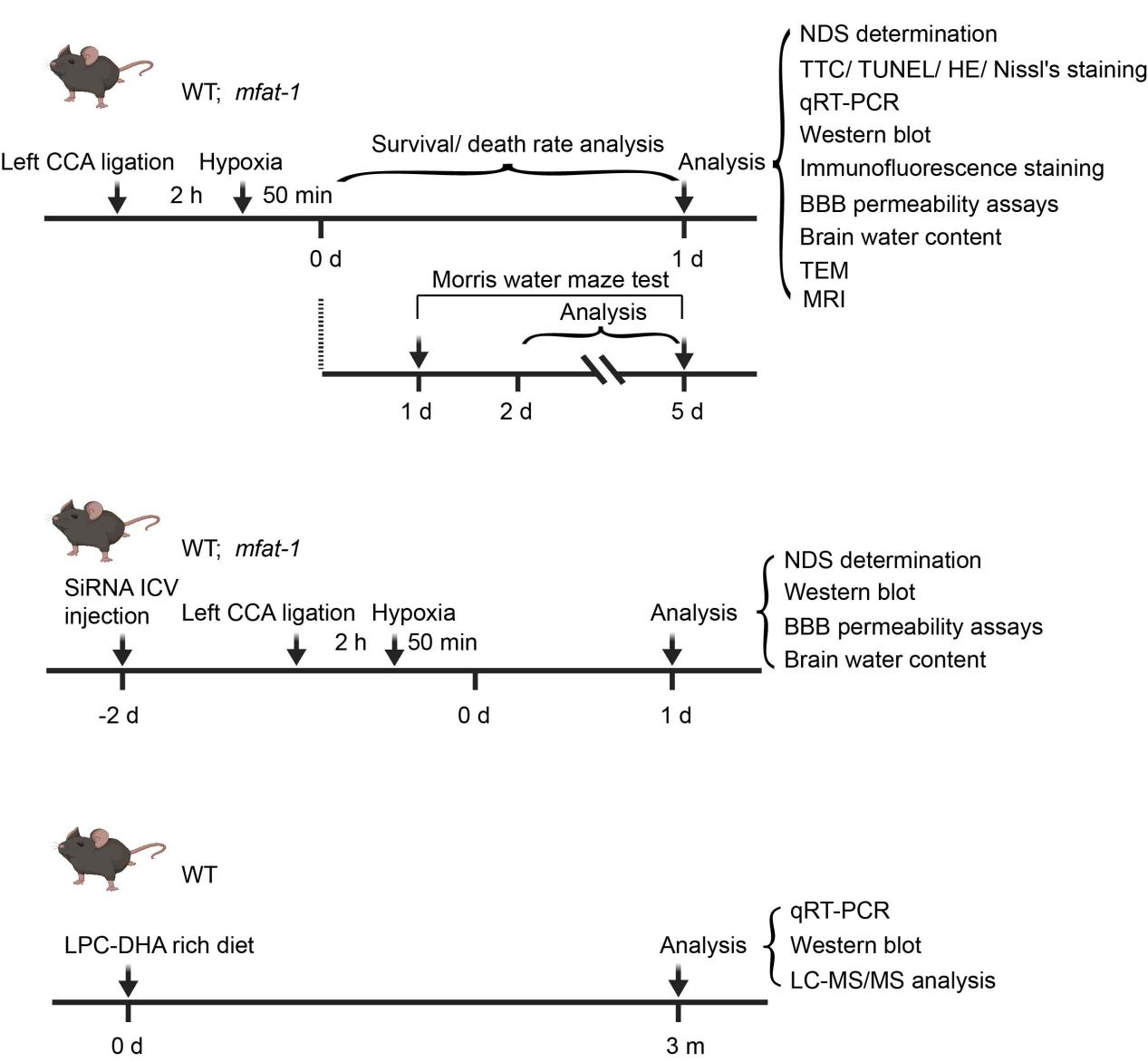


**Fig. S7** A timetable graphic outline of the whole study design. [Illustration created with BioRender]


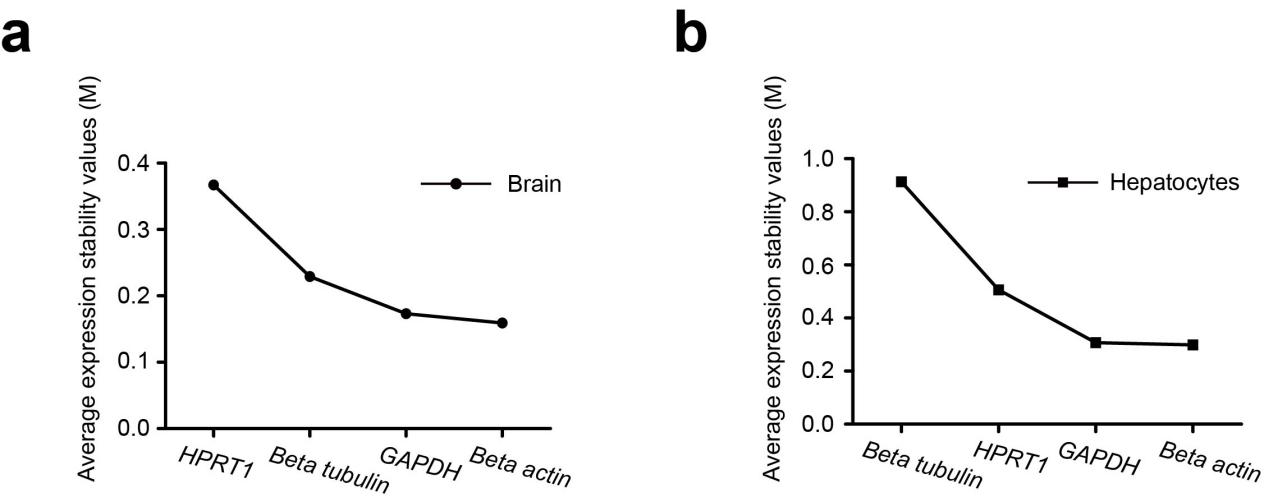


**Fig. S8** *Beta actin* was the most stable reference gene in the brain tissues and hepatocytes of mice. **a and b** Average expression stability values (M) of candidate reference genes (*HPRT*, *beta tubulin*, *GAPDH* and *beta actin*) in the mice's brain tissues (WT group (n = 14) and *mfat-1* group (n = 10)) (**a**) and hepatocytes derived from different mice (control siRNA group (n = 10) and *Mfsd2a*-siRNA group (n = 8)) (**b**) via qRT-PCR analyzed by NormFinder. M is inversely proportional to gene stability.


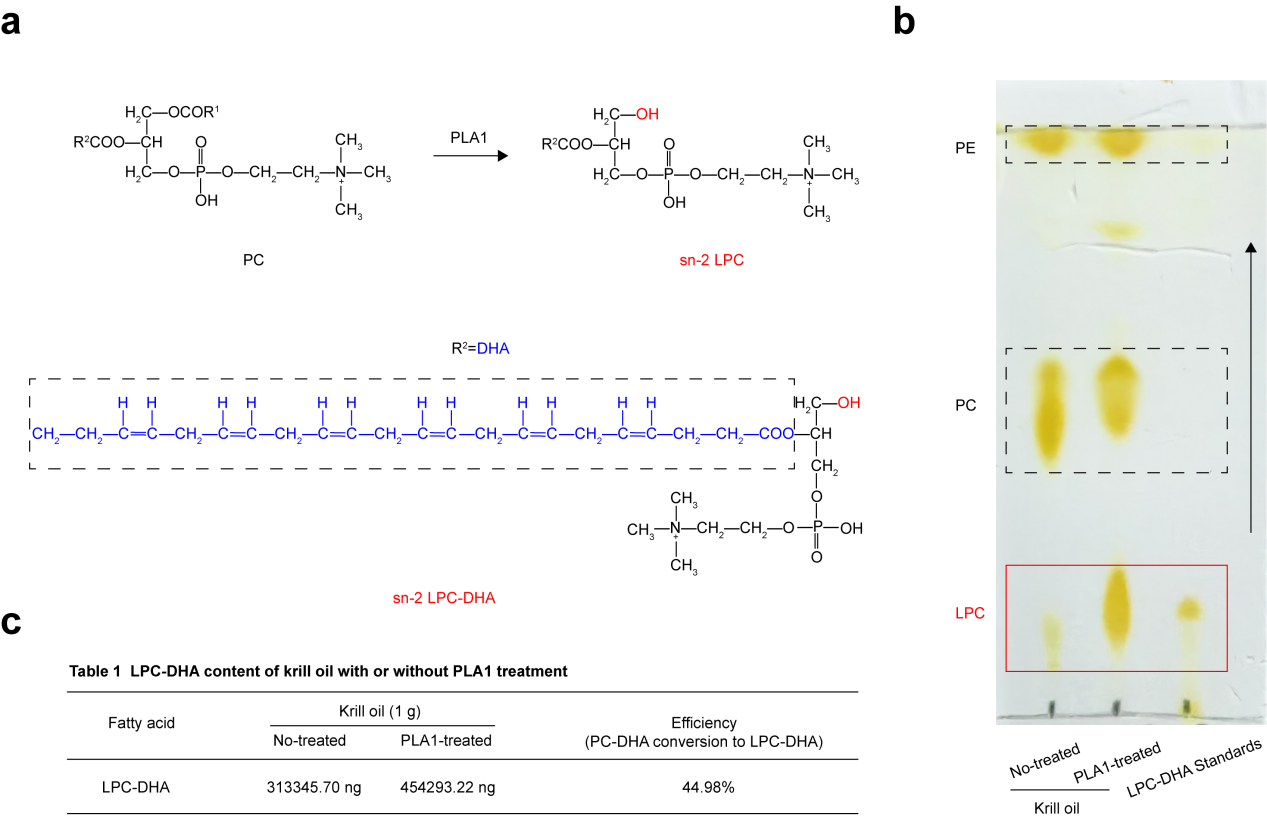


**Fig. S9** Hydrolysis mechanism of PLA1 and TLC results. **a** Up: the hydrolysis mechanism of PLA1, that catalyzes the hydrolysis of the acyl group from position 1 of PC to yield sn-2 LPC. R^1^ and R^2^ represented the group at position 1 and position 2, respectively. Down: the structural formula of sn-2 LPC-DHA. **b** TLC results in no-treated krill oil, PLA1-treated krill oil and LPC-DHA Standards (Cayman). Original krill oil mainly showed three spots: phosphatidyl ethanolamine (PE), PC and LPC. PC was the main component according to the chromogenic area. The fatty acid content of LPC-form was significantly increased in PLA1-treated krill oil, including LPC-DHA. The spots of TLC at the same position between different groups represent the fatty acids in the same form of phospholipids, not only one substance. The black arrow represents the moving direction of the developing solvent. **c** The LPC-DHA content of 1 g krill oil with or without PLA1 treatment was analyzed by LC-MS/MS. The conversion efficiency of PC-DHA to LPC-DHA was significantly increased after PLA1 treatment.
